# Machine learning-based quantitative structure–activity relationship model for antibiotic prediction and discovery

**DOI:** 10.1371/journal.pdig.0001593

**Published:** 2026-08-13

**Authors:** Jiratchaya Nakbang, Chonthicha Arbsuwan, Santitham Prom-on, Peerut Chienwichai

**Affiliations:** 1 Princess Srisavangavadhana Faculty of Medicine, Chulabhorn Royal Academy, Lak Si, Bangkok, Thailand; 2 Department of Computer Engineering, Faculty of Engineering, King Mongkut’s University of Technology Thonburi, Thung Khru, Bangkok, Thailand; 3 Research Center on Clinical and System Microbiology (RCSyM), Chulabhorn Royal Academy, Lak Si, Bangkok, Thailand; Yonsei University College of Medicine, KOREA, REPUBLIC OF

## Abstract

Antimicrobial resistance (AMR) is a critical global health problem that has become increasingly alarming in recent years. The discovery of new antibiotics is one approach for alleviating AMR, however, screening for novel drugs is time consuming and expensive. To accelerate antibiotic discovery, the integration of machine learning algorithms with Quantitative Structure–Activity Relationship (QSAR) calculations could provide a rapid solution. Thus, this study combines a QSAR model and machine learning algorithms to predict antibacterial activities of potential novel drugs based on chemical information. Information on compounds that are reportedly active and inactive against bacteria was downloaded from the PubChem database and manually curated to create positive and negative datasets. The decision tree (DT), support vector machine (SVM), and naïve Bayesian (NB) algorithms were employed to predict the antibacterial activities of chemical compounds from their Simplified Molecular Input Line Entry System (SMILES) information. The models were then evaluated quantitatively and tuned. DT and SVM exhibited comparable predictive performance and outperformed the NB model, achieving accuracy, precision, sensitivity, and AUC-ROC values exceeding 0.90. DT was chosen for further analysis because of its simplicity and effectiveness. This revealed that descriptors relating to the electrotopology and β-lactam structures of compounds were the top contributors to model predictability. The model was then further tested against different classes of antibiotics and achieved high accuracy in all classes. The model is freely available as a web application at: https://antibacterial-predictor-model-ocogzqyibervrqb7trvfev.streamlit.app/.

## Author summary

Antimicrobial resistance (AMR) is a serious global health challenge, with its incidence steadily increasing while the rate of new antibiotic development continues to decline. The process of antibiotic discovery is costly and time-consuming, making it difficult to keep pace with the growing threat of resistant pathogens. To address this urgent need, computer-aided drug discovery has emerged as a promising solution. In this study, we developed a machine learning model for antibiotic prediction. Our model demonstrated strong performance, achieving over 90% accuracy, precision, and sensitivity, effectively distinguishing compounds with antibacterial properties from those without. Importantly, the model’s decision-making process is interpretable, enhancing the reliability of its predictions. Furthermore, we have packaged the model into a web application, making it accessible for broader use. This tool aims to accelerate antibiotic discovery, particularly for researchers without a background in data science, and contributes to the global effort to combat AMR.

## Introduction

Antimicrobial resistance (AMR) poses an increasing threat to global health. Globally, in 2021, 4.71 million deaths were associated with bacterial AMR. AMR-related mortality among adults –particularly the elderly–is projected to rise by over 80% by 2050, reaching 8.22 million deaths [1]. Economic losses due to AMR are estimated to reach between US$300 billion and US$1 trillion by 2050, primarily driven by increasing medical and nursing care costs [2]. Substantial efforts have been expended to ameliorate the AMR crisis, including antimicrobial stewardship practices, rigorous surveillance systems, and the development of novel drugs against such pathogens [3]. Antimicrobial stewardship involves with the process of optimizing the use of antimicrobial agents, aiming to reduce the development of AMR organisms, especially bacteria. For antimicrobial stewardship to be successful, collaboration among many groups, such as medical staff, veterinary personnel, and patients, is required, which poses a challenge in implementation [4, 5]. Meanwhile, surveillance systems for AMR are essential for understanding the burden of AMR and preparing appropriate countermeasures [6]. Such medical crisis monitoring has been conducted in many countries worldwide, including the United Kingdom, Japan, and India, but information on the types and prevalence of AMR bacteria are still lacking in many regions [7–9]. Finally, the development of new antimicrobial agents is another approach to combat resistant pathogens; however, this process is time consuming and has a low success rate [10]. Many methods have been applied to overcome these challenges in the discovery of novel antimicrobial compounds.

Classical antimicrobial drug development usually relies on discovering new active compounds from natural sources. For example, Choi *et al.* discovered natural antimicrobial products (NP) possessing broad-spectrum antimicrobial activities from the filtered broth of the mold *Aspergillus oryzae.* Their NP showed potent bactericidal activity against several bacteria, including *Staphylococcus aureus*, *Listeria monocytogenes*, *Salmonella typhimurium*, *Klebsiella pneumonia, Pseudomonas aeruginosa,* and *Escherichia coli.* Interestingly, the NP also showed antifungal activity against *A. fumigatus, Candida albicans*, and *Penicillium* spp. [11]. Liu *et al.* studied the chemical composition of the herb *Artemisia absinthium* and identified a number of bactericidal compounds against groups of gram-positive and gram-negative bacteria. They found that salicylic acid, caffeic acid, casticin, and 3,4-dicaffeoylquinic acid could effectively inhibit the growth of *S. aureus, Streptococcus epidermidis, Bacillus subtilis, B. cereus, P. aeruginosa, E. coli, K. aerogenes, K. pneumoniae*, and *C. albicans* and proposed these compounds as candidates for antibiotic development [12]. Although many potential antibiotics have been identified using classical approaches, challenges remain regarding cost effectiveness, the duration from discovery to clinical application, and the low success rate. To improve the efficiency of antibiotic screening, computer algorithms have been applied, with the Quantitative Structure–Activity Relationship (QSAR) model being one of the most popular choices. This is a mathematical modeling method used to calculate the molecular descriptors of chemical compounds, which can then be used to predict their biological activities [13]. Notably, many studies have proposed compounds that potentially exhibit bactericidal properties using the QSAR method for screening. For example, Ghasedi *et al.* performed QSAR modeling on 39 quinolone-triazole derivative compounds with the aim of identifying novel drugs against *S. aureus* and *P. aeruginosa.* By applying a multiple linear regression model to the chemical descriptors generated from these derivatives, the key descriptors contributing to the antibacterial activities of the compounds were identified and used to modify their structures to enhance potency [14]. Cloete *et al.* also used QSAR modeling, together with other computational algorithms, to screen for an inhibitor of nicotinamide-nucleotide adenylyl transferase, a vital enzyme for *Mycobacterium tuberculosis.* With the application of QSAR, they narrowed down their targets from 155 to 5 compounds for further tests, among which novobiocin sodium salt showed profound activity against this pathogen [15]*.*

Most QSAR studies have used regression-based models, such as multiple linear regression and curvilinear regression, to predict antibacterial properties [14,16,17]. With the advancement of artificial intelligence and machine learning, various algorithms, such as Random Forest (RF) and Support Vector Machine (SVM), have been utilized to enhance QSAR-based antibiotic discovery. Machine learning algorithms have demonstrated advantages in drug discovery by effectively handling high-dimensional data, processing non-linear relationships, and identifying key features from large datasets. These advantages make them well-suited for classifying complex datasets, such as chemical structures and molecular relationships [18–20]. Compared to the classical drug discovery pipeline, a machine learning-based QSAR approach offers a more efficient approach, enabling the screening of a larger number of potential antibacterial compounds in less time and with fewer resources [13]. For example, Wani and Roy explored the use of machine learning to identify potential anti-tubercular agents from a dataset of 776 compounds. By employing decision tree (DT) models, they achieved a strong predictive score of 0.77. They suggested that interpreting anti-tubercular agents based on results from multiple models would enhance reliability and accuracy [21]. In a study by Zhang *et al.*, the RF algorithm was used to screen 955 pleuromutilin derivatives for antibacterial activity against *S. aureus* and methicillin-resistant *S. aureus*. Their model achieved an accuracy of 0.80, with *in vitro* assays confirming its performance in identifying new drugs for killing these bacteria [22]. Additionally, in a study by Boulaamane *et al*., five algorithms—RF, SVM, k-nearest neighbors (KNN), Gaussian naïve Bayes, and convolutional neural networks (CNN)—were used to classify bioactive compounds against *Acinetobacter baumannii*. Their prediction model achieved an area under the receiver operating characteristic curve (AUC-ROC) of 0.96, indicating high classification power [23]. While the results from these studies are promising, most have focused narrowly on extracting chemical descriptors from a specific group of compounds, either antibiotic derivatives or compounds targeting a single species of bacteria. This limits the generalizability of the model and creates difficulties in discovering novel antibiotics from a large set of compounds. To overcome this limitation, this study aims to develop a machine learning–based QSAR model for predicting antibacterial activity from chemical information. Data on antibacterial compounds were obtained from the PubChem database, and chemical descriptors were generated using the RDKit package. Machine learning algorithms, including DT, SVM, and naïve Bayesian (NB), were trained and fine-tuned to achieve optimal performance. Afterward, the best model from our study was converted into a Python package and deposited in a public repository for use by the scientific community. These findings may contribute to accelerating antibiotic discovery efforts aimed at addressing the escalating global threat of AMR.

## Materials and methods

### Data source and acquisition

Data on compounds with and without antibacterial activity were retrieved from the PubChem database on Oct 30^th^, 2023 [24]. To compile antibacterial compounds, the keywords “antibacterial” and “antibiotic” were used for the search. Compounds were manually curated for antibacterial activity. Compounds annotated as active were classified as antibacterial, while compounds without explicit activity annotation but with reported minimum inhibitory concentration (MIC) values were also classified as antibacterial. The data on compound name, molecular formula, canonical SMILES, InChIKey, and activity were downloaded from the database and then considered the positive dataset. For compounds without antibacterial activity, the keywords “antacid,” “antihistamine,” “analgesic,” “chelating,” “cardiovascular,” “contraceptive,” and “sedative” were applied to obtain drugs with different structures. Similarly, compounds with antibacterial activity were excluded. Compounds without antibacterial activity were downloaded and randomly selected to match the number in the positive dataset and then considered as the negative dataset.

### Feature extraction

Compounds in the positive and negative datasets underwent a feature extraction process using the RDKit library [25]. From RDKit, 217 chemometric descriptors were generated based on the canonical SMILES data of each drug, covering a vast range of features of substances, such as physicochemical, topological, constitutional, and electronic properties, as well as molecular diversity. Features with infinite values or no variance were excluded. Furthermore, Z-score normalization was performed to reduce the variability between the descriptors.

### Model development and hyperparameter tuning

Chemical descriptors from both positive and negative sets were divided into 80:20 ratios for model training/validation and testing. Three machine learning algorithms were employed: DT, SVM, and NB. These models were selected because they are commonly used in QSAR studies and offer complementary strengths. DT was chosen for its interpretability, SVM for its effectiveness in high-dimensional feature spaces, and NB as a simple probabilistic baseline, allowing a balanced and systematic comparison of different modeling approaches [26]. The parameters of each model were adjusted accordingly to achieve optimal model performance. For all algorithms, the value for a random state was set to 42. In DT, entropy was chosen as a criterion for splitting nodes. In the SVM, the probability parameter was set to TRUE, and the data were linearly separated using the kernel function.

To increase the predictive performance of the algorithm, the model that showed the best performance was subjected to hyperparameter tuning. After the first round of model training, all models were evaluated for their performance with a validating dataset. The best model was further tuned based on the following parameters: Max_depth = 1–11; Max_features = {None, sqrt, log2}, Min_samples_leaf = {1, 2, 4}; Min_samples_split = {2, 5, 10} Moreover, a grid search and K-fold cross-validation were applied to enhance the performance of hyperparameter tuning [27].

### Feature selection

After completing the hyperparameter tuning process, we proceeded to enhance the model by selecting the most influential descriptors contributing to its predictive performance. To achieve this, we calculated the feature importance value of each descriptor based on the trained DT model. Descriptors with an importance value of zero were considered non-contributory and were excluded from further analysis. The remaining descriptors were ranked according to their importance values, and the top 20 most influential descriptors were selected for developing an optimized model using the tuned parameters identified earlier.

To evaluate the impact of this feature selection step, we compared the performance of the newly developed model (using only the top 20 descriptors) with that of the original model that included all available descriptors. This comparison aimed to assess whether reducing the number of descriptors would preserve, or potentially even enhance, predictive performance while improving model efficiency and interpretability. In addition, SHapley Additive exPlanations (SHAP) values of the top descriptors were calculated to explain how the descriptors impacted model prediction [28].

### Model evaluation

Four parameters were used to evaluate model performance in all processes: accuracy, precision, sensitivity, and AUC-ROC.

- Accuracy = $\frac{True\;positive\;+\;True\;negative}{True\;positive\;+\;False\;positive\;+\;True\;negative\;+\;False\;negative}$
- Precision = $\frac{True\;positive}{True\;positive\;+False\;positive}$
- Sensitivity = $\frac{True\;positive}{True\;positive\;+\;False\;negative}$

For AUC-ROC, the AUC was measured from the curve between the true positive rate and the false positive rate, which were calculated using the following formulas:

- True positive rate = $\frac{True\;positive}{True\;positive\;+\;False\;negative}$
- False positive rate = $\frac{False\;positive}{False\;positive\;+\;True\;negative}$

**Fig 1:**
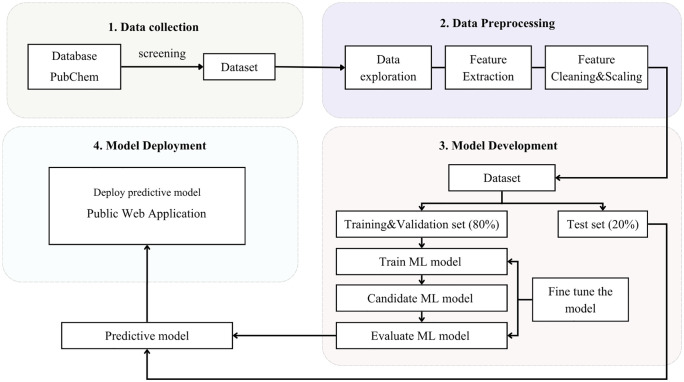
Flowchart of the study.

In addition, the positive testing dataset was stratified into nine classes of antibiotics based on a previous report [29]. The fully developed model was then tested on data from each antibiotic class to evaluate its performance across antibiotics with different mechanisms of action. The full model developed in this work can be found as a web application on Streamlit server [30]. A flow chart of this work is presented in Fig 1.

## Results

To develop machine learning models for antibacterial prediction, an initial search of the PubChem database was performed to collect data. This search, specifically targeting antibacterial drugs, yielded 6,291 results. With manual curation, there were 2,095 drugs that showed activity against bacteria and for which the minimal inhibitory concentration was available. This was considered the positive dataset. To ensure the assembly of a negative dataset with various drug structures, seven groups of drugs were chosen, and their data were processed. A total of 3,738 drug structures were obtained, of which 2,095 were randomly selected to constitute the negative dataset, thus avoiding an imbalance between the positive and negative datasets.

**Fig 2:**
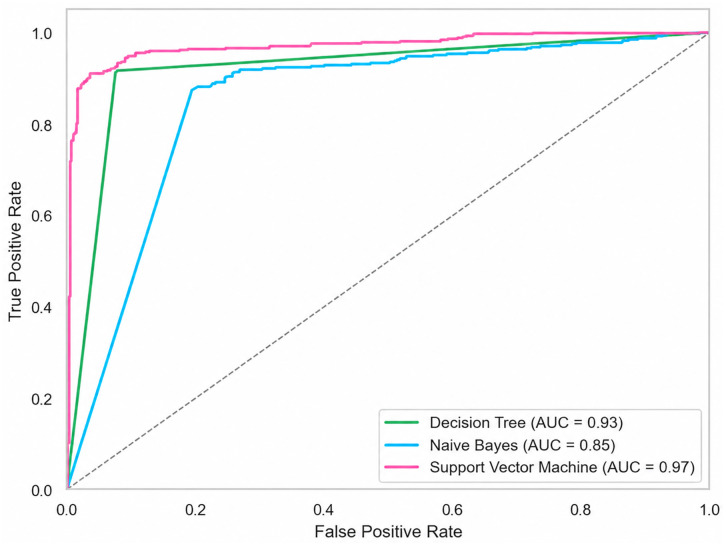
Comparison of AUC-ROC curves for the DT, SVM, and NB models.

Of the 217 chemometric descriptors, 5 had zero or infinite variance; therefore, these were excluded. The remaining 212 descriptors were used for model development. The chemometric descriptors of 4,190 drugs were divided into two groups: 80% as a set for training and validation and 20% as a testing set (S1 Table). In our study, three machine learning algorithms were used to classify the antibacterial properties of the compounds: DT, SVM, and NB. Interestingly, DT and SVM showed similar performance, with accuracy, precision, sensitivity, and AUC-ROC exceeding 0.90 (Table 1, Fig 2). Meanwhile, NB achieved notably lower accuracy and precision, at 0.71 and 0.65, respectively. The above results suggest that DT and SVM are highly effective in predicting the antibacterial properties of compounds. However, only the DT algorithm was chosen for further development because of the explainable nature of the model, which would be beneficial for understanding the descriptors that play vital roles in antibiotic classification.

**Table 1:** Performance comparison of the DT, SVM, and NB models.

Model performance	Accuracy	Precision	Sensitivity
Decision tree	0.93	0.92	0.93
Support vector machine	0.92	0.92	0.91
Naïve Bayesian	0.71	0.65	0.94

**Fig 3:**
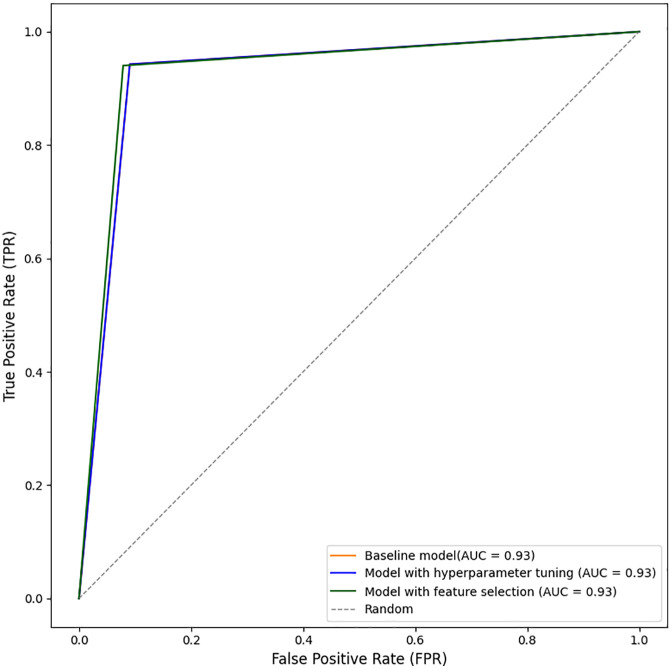
Comparison of AUC-ROC curves for the DT model performance: baseline model, model with hyperparameter tuning, and model with feature selection.

Hyperparameter tuning did not improve the performance of the DT model. In fact, its accuracy and sensitivity decreased slightly by 0.01 and 0.02, respectively (Table 2). Subsequently, feature selection was performed to reduce the number of chemometric descriptors used for model development. The top 20 descriptors that most influenced the decision of the DT model were selected for analysis (Table 3). The DT model developed with these 20 descriptors exhibited similar performance to the model created with 212 descriptors (Table 2, Fig 3), suggesting that these 20 descriptors have a particularly significant impact on the antibacterial classification of the models. The fully developed model achieved good performance with accuracy, precision, sensitivity, and AUC-ROC of 0.92, 0.92, 0.91, and 0.93, respectively.

**Table 2:** Comparison of DT model performance: baseline model, model with hyperparameter tuning, and model with feature selection.

Model performance	Accuracy	Precision	Sensitivity
Baseline model	0.93	0.92	0.93
Model with hyperparameter tuning	0.92	0.92	0.91
Model with feature selection	0.92	0.92	0.91

**Table 3:** Feature importance value of top 20 descriptors that influenced on DT model, before and after feature selection process.

Descriptor	Descriptor type*	Descriptor explanation [25].	Feature importance value	
**Before selection**	**After selection**
EState_VSA1	T	MOE-type descriptors using EState indices and surface area contributions. (-inf ≤ x < -0.39)	0.320	0.320
BCUT2D_CHGLO	MD	Lowest eigenvalue weighted by Gasteiger charges	0.135	0.150
VSA_EState6	T	MOE-type descriptors using EState indices and surface area contributions. (6.00 ≤ x < 6.07)	0.073	0.087
MaxAbsEStateIndex	T	Maximum absolute EState index	0.050	0.068
fr_lactam	C	Number of beta lactams	0.056	0.056
BCUT2D_MRHI	MD	Highest eigenvalue weighted by Crippen MRR	0.032	0.042
TPSA	P	Topological surface area	0.025	0.033
BalabanJ	T	Balaban’s J value for a molecule.	0.022	0.032
SPS	T	Spacial Score	0.020	0.029
VSA_EState4	T	MOE-type descriptors using EState indices and surface area contributions. (5.41 ≤ x < 5.74)	0.024	0.025
VSA_EState8	T	MOE-type descriptors using EState indices and surface area contributions. (6.45 ≤ x < 7.00)	0.012	0.024
BCUT2D_MWHI	MD	Highest eigenvalue weighted by atomic masses.	0.012	0.023
fr_lactone	C	Number of cyclic esters (lactones)	0.020	0.020
MaxEStateIndex	T	Returns a tuple of EState indices for the molecule.	0.025	0.019
SlogP_VSA3	P	MOE logP VSA Descriptor 3 (-0.20 <= x < 0.00)	0.008	0.014
NumHeterocycles	C	Number of Heterocycles	0.013	0.014
PEOE_VSA9	P	MOE Charge VSA Descriptor 9 (0.05 <= x < 0.10)	0.010	0.013
BCUT2D_MRLOW	MD	Highest eigenvalue weighted by atomic masses.	0.006	0.013
BCUT2D_LOGPLOW	MD	Highest eigenvalue weighted by atomic masses.	0.007	0.010
SlogP_VSA2	P	MOE logP VSA Descriptor 2 (-0.40 <= x < -0.20)	0.008	0.009

* P = Physico-chemical property, T = Topological property, C = Constitutional property, E = Electronic property, MD = Molecular diversity.

Afterward, the feature analysis was performed to understand the process of model classification. With the baseline DT model, the top 20 descriptors that most markedly affected the model predictions were analyzed. Topology was the descriptor property that most influenced the decision-making process of the algorithm, with a total importance value of 0.546. EState_VSA1, BCUT2D_CHGLO, VSA_EState6, fr_lactam, and MaxAbsEStateIndex were the top 5 descriptors in terms of the feature importance values of the DT model before feature selection (Table 3).

**Fig 4:**
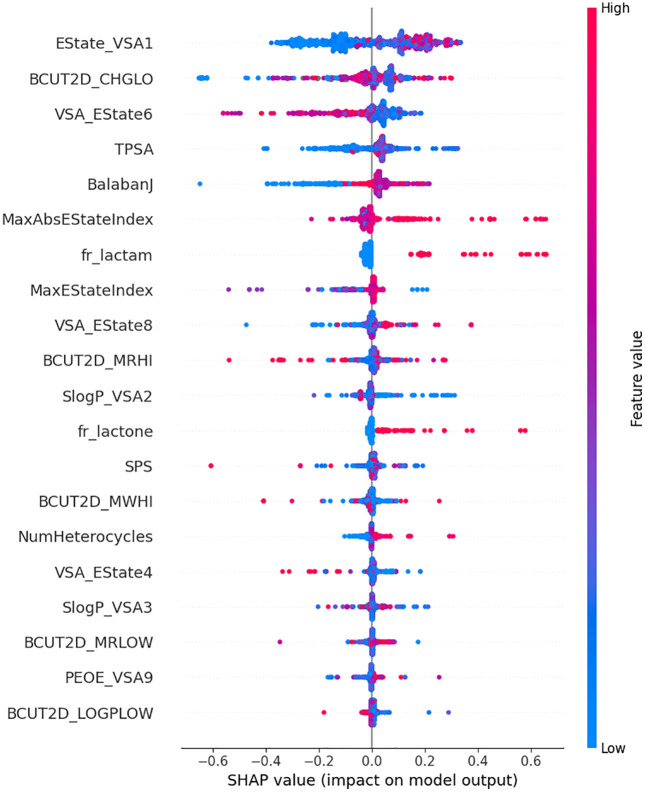
Shapley plot depicting the SHAP value of each descriptor for model development after feature selection.

**Fig 5:**
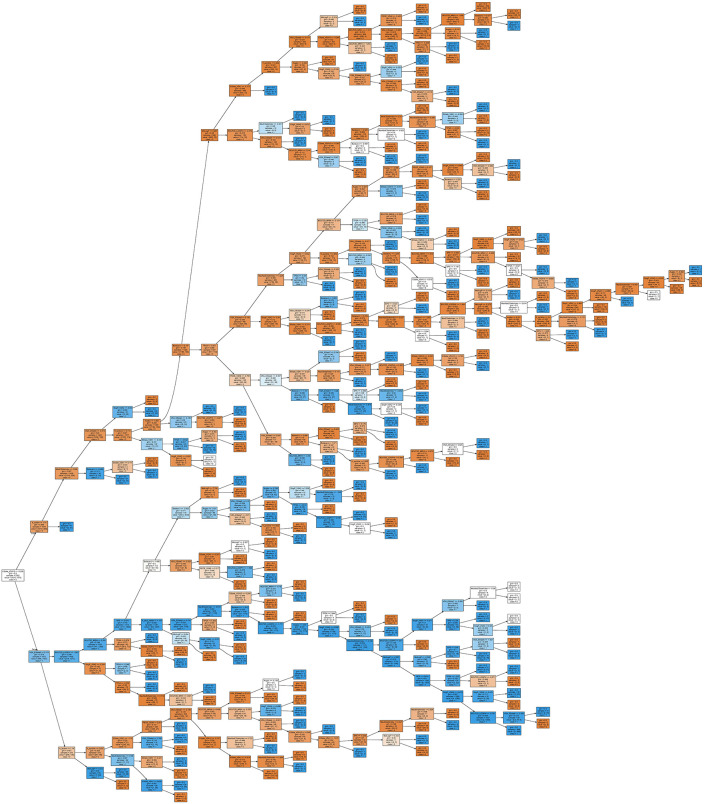
Decision-making process of the DT model after feature selection.

Following the feature selection process, the feature importance of each descriptor was re-calculated to observe the impact of the descriptors on the new model. Topological descriptor properties were still the type that most markedly influenced the model results. The top five descriptors were largely unchanged after feature selection, except that MaxAbsEStateIndex became more contributory than fr_lactam. On the other hand, several descriptors exhibited distinct patterns in the Shapley plot, indicating their contributions to the model’s predictions and facilitating interpretation of the decision-making process. The compounds with low EState_VSA1 were unlikely to possess antibacterial activity. In contrast, compounds with low VSA_EState6 values tended to exhibit more antibacterial activity than those with high values. Interestingly, fr_lactam displayed distinct clustering of compounds with low and high descriptor values, with higher values generally associated with increased antibacterial activity (Fig 4). The decision process of the DT model is shown in Fig 5.

Beyond understanding the contribution of individual descriptors, it is also important to examine how the model performs across diverse antibiotic classes. Testing the fully developed model on different classes of antibiotics revealed that it achieved an accuracy of over 0.8 for all classes (S1 Fig). This finding suggests that the model can reliably predict molecules with diverse structures and mechanisms of action with high performance. This model is freely available as a web application for broader usage (https://antibacterial-predictor-model-ocogzqyibervrqb7trvfev.streamlit.app/). The input for this tool is canonical SMILES of the compounds of interest, after which the model calculates all the required features and classifies the compounds as active or inactive. Users can easily interpret the results and use them for further antibiotic testing *in vitro.*

## Discussion

Antimicrobial resistance poses a global threat that cannot be overlooked. The discovery of new antibacterial drugs is a promising solution for alleviating this threat, but screening for active compounds remains a bottleneck in this process. This study attempted to accelerate this process by developing a machine learning–based QSAR model for the prediction of the antibacterial effects of compounds. In terms of its performance, the QSAR model achieved values exceeding 0.9 for accuracy, precision, sensitivity, and AUC-ROC. The model was also tested on different classes of antibiotics, achieving high accuracy across almost all classes. This model is freely available in the form of a web application and requires only canonical SMILES of the compounds for prediction. The algorithm indicated that EState_VSA1, BCUT2D_CHGLO, VSA_EState6, fr_lactam, and MaxAbsEStateIndex were the chemical features of compounds that most markedly influenced their biological activity against bacteria.

EState_VSA1 was the descriptor that demonstrated the highest feature importance, with a value of 0.320 (Table 3). EState_VSA1 is a molecular operating environment (MOE)-type descriptor based on electrotopological state indices and surface area calculations, with values ranging between -inf and -0.39 [25]. Similarly, VSA_EState6, a chemometric descriptor, had the third highest feature importance, with a value of 0.087 (Table 3). This MOE–type descriptor is calculated using electrotopological state indices and surface area, and its value ranges between 6.00 and 6.07 [25]. Notably, Wang *et al.* studied the analogs of an antibiotic—polymyxin—and their effects on gram-negative bacteria. Using descriptor analysis, they found that VSA_EState6 was the descriptor with the second highest feature importance value. They also identified many important descriptors similar to those in our study, such as VSA_Estate8, BCUT2D_CHGLO, and BalabanJ. However, they did not provide an explanation of the mechanism by which these features contribute to the function of antibiotics [31]. Rzycki *et al.* reported the same descriptors as in our study that influenced the antibacterial properties of molecules. They selected a group of antibiotics and investigated their clustering and feature importance using machine learning approaches. Their findings identified VSA_Estate6 and MaxEStateIndex as the most influential descriptors, accounting for more than 35% of the contributions [32]. Interestingly, Cooper *et al.* evaluated the antibacterial activities of drugs on *E. coli* and *P. aeruginosa* and used the RF algorithm to identify key contributors to their functions. They found that descriptors related to charges and van der Waals surface area (VSA) regions were the factors that predominantly determined the activity of compounds against these bacteria, similar to the findings in our study. They proposed that drugs with these features could limit efflux out of bacterial cells and that they could be retained in the cytoplasm more effectively than other drugs [33]. There are no reports currently linking the mechanism of antibiotic action to the EState_VSA1 and VSA_EState6 descriptors. However, the electrotopological features of compounds have been associated with protein binding affinity [34]. Based on this, we hypothesize that these descriptors may influence the mechanism of action of antibiotics that require protein binding, such as fluoroquinolones that bind to target DNA gyrase [35].

The descriptor fr_lactam represents the number of β-lactam structures found in molecules [25]. β-Lactam is a four-membered heterocyclic amide ring, which is the core structure of many antibiotics, such as penicillin, cephalosporin, and carbapenem (referred to as β-lactam antibiotics) [36, 37]. β-lactam antibiotics are one of the three largest classes of antibiotics that target the cell wall of gram-positive bacteria [38]. β-lactam acts as a pseudosubstrate for bacterial transpeptidase, the key enzyme for cell wall synthesis, causing the inhibition of transpeptidase activity and bactericidal effects [36, 37]. The presence of a β-lactam ring in molecules is a strong indicator of antibacterial properties, particularly against gram-positive bacteria, resulting in the fr_lactam descriptor having a high feature importance value. This finding is consistent with the model’s performance on β-lactam antibiotics, for which it achieved an accuracy of 0.938 (S1 Fig). Our feature analysis findings show that the decision process of our DT model is reliable and based on prior information.

The DT model was chosen for the classification of compounds with and without antibacterial properties based on its high performance and the interpretability of the decision-making algorithm. For machine learning–based QSAR models, many algorithms have been employed to achieve excellent antibacterial prediction performance for various groups of bacteria. For example, Wang *et al.* conducted computational experiments for predicting compounds targeting *A. baumannii, E. coli, K. pneumoniae, P. aeruginosa*, and gram-negative bacteria in general. They developed a QSAR model based on chemometric descriptors of polymyxin, the antibiotic of choice for treating gram-negative bacterial infections. With graph convolutional networks and CatBoost-based machine learning, the accuracy was 0.80, 0.75, 0.70, 0.78, and 0.80 for *A. baumannii, E. coli, K. pneumoniae, P. aeruginosa,* and gram-negative bacteria in general, respectively [31]*.* Boulaamane *et al.* and Koh *et al*. also studied bacteria from among these groups [23,39]. Specifically, Boulaamane *et al*. used five algorithms to discover bactericidal substances targeting *A. baumannii.* They found that CNN outperformed other algorithms, with accuracy, precision, and AUC-ROC exceeding 0.9 [23]. Similarly, Koh *et al.* used eight algorithms to create models for predicting inhibitors of *las* quorum sensing, a drug target for treating *P. aeruginosa* infection*.* They found that multilayer perceptron was the best algorithm, with an accuracy of 0.93 and an AUC-ROC of 0.92 [39]. Focusing on vital bacterial enzymes is another approach for developing models for antibiotic discovery. For example, Aniceto *et al.* developed a QSAR model by targeting urease, a vital enzyme in some bacteria, including *Helicobacter pylori* and *Proteus mirabilis.* It was anticipated that the inhibition of this enzyme would profoundly affect the viability of these pathogens. They used three machine learning models to predict the activities of the compounds: RF, DT, and extreme gradient boosting. RF was found to be the best model and achieved a precision and sensitivity of approximately 0.8. Meanwhile, only one study has focused on a QSAR model for gram-positive bacteria [40]. Zhang *et al.* employed an RF algorithm to predict compounds effective against anti-*S. aureus* and anti-methicillin-resistant *S. aureus.* In this approach, the model precision and sensitivity were 0.80 and 0.83, respectively [22].

Most QSAR studies have focused on specific bacterial targets, which theoretically enhance model performance due to the specialized data used in model development. However, this approach may not be suitable for screening new compounds for general antibacterial activity, as it would be time consuming to screen multiple models to identify the target pathogen. To date, only two studies have used machine learning algorithms to predict the antibacterial properties of compounds against bacteria in general, without specifying a species. The first of these studies, Yang *et al.*, employed support vector classification (SVC), KNN, and C4.5 DT to classify 230 compounds with and 381 compounds without antibacterial properties. The SVC algorithm showed the best performance after the feature selection process, with an accuracy of 0.98 [41]. Subsequently, Pereira *et al.* used a classification tree, RF, and SVM to classify compounds into antitumor and antibiotic drugs. In terms of antibiotics, the numbers of active and nonactive compounds were 642 and 1104, respectively. RF achieved the highest sensitivity of 0.83 [42]. Unfortunately, neither of these studies provided the source code of the model or developed it into a package, preventing the use of the model elsewhere. Our work generated a machine learning–based QSAR model with the goal of generalizing the prediction of antibacterial compounds. Our model achieved comparable performance for both general and specific target bacteria (Table 4). The model was then developed into a web application. The fully trained model is easy to use and requires only the canonical SMILES of compounds to predict their activity. The web application is freely available, which should accelerate the drug discovery process to combat the threat of AMR.

**Table 4:** The comparison of performances of machine learning-based QSAR studies with this study.

No.	Study	Target	Algorithm used	Best algorithm	Performance
1	This study	General bacteria	DT, SVM, NB	DT	Accuracy = 0.92
					Precision = 0.92
					Sensitivity = 0.91
					AUC-ROC = 0.93
2	Yang *et al*. [41]	General bacteria	SVC, KNN, and C4.5DT	C4.5DT	Accuracy = 0.98
					Sensitivity = 0.96
3	Pereira, *et al.* [42]	General bacteria	Classification tree, RF, and SVM	RF	Sensitivity = 0.83
4	Wang, *et al.* [31]	*A. baumannii, E. coli, K. pneumoniae, P. aeruginosa*, and general gram negative bacteria	Graph convolutional network	Graph convolutional network	Accuracy = 0.80
					Precision = 0.77
					Sensitivity = 0.63
					AUC-ROC = 0.86
5	Boulaamane, *et al*. [23]	*A. baumannii*	RF, SVM, KNN, Gaussian Naive Bayes, and CNN	CNN	Accuracy = 0.90
					Precision = 0.95
					Sensitivity = 0.83
					AUC-ROC = 0.96
6	Koh, *et al.* [39]	*P. aeruginosa*	Bayes Net, Multinomial Naïve Bayes, Logistic Regression, Multilayer Perceptron, SVM, KNN, KStar, RF	Multilayer Perceptron	Accuracy = 0.93
					AUC-ROC = 0.92
7	Aniceto, *et al.* [40]	*H. pylori* and *P. mirabilis*	RF, DT, Extreme gradient boosting	RF	Precision = 0.81
					Sensitivity = 0.79
8	Zhang, *et al.* [22]	*S. aureus* and methicillin-resistant *S. aureus*	RF	RF	Precision = 0.80
					Sensitivity = 0.83

The key strength of the present study lies in the development of a user-friendly prediction tool for antimicrobial activity based solely on SMILES representations, implemented as a web application to facilitate broad accessibility. The model demonstrates strong predictive performance against bacterial targets, making it particularly suitable for primary screening of antimicrobial compounds. Importantly, the incorporation of interpretable machine-learning approaches enhances the transparency and reliability of the predictions, allowing users to better understand the underlying decision process. Moreover, the tool is publicly available, supporting general use by the scientific community and aiming to accelerate early-stage antimicrobial discovery as a rapid response to the growing threat of AMR. Nevertheless, several limitations of the current approach should be acknowledged. Firstly, the model development in this study relied based on compounds with similar structure or substructure would have similar biochemically function. With this fundamental, we predicted compounds those have similar structures with antibiotics, or antibiotic-like molecules, rather than molecules with direct antibacterial properties. To overcome this limitation, we encouraged the biological validation of our model prediction before further testing. Specifically, compounds predicted as active will undergo *in vitro* antibacterial assays, such as MIC determination against representative bacterial strains, to confirm biological activity. Promising candidates may then be further evaluated for mechanism of action and cytotoxicity profiling. Secondly, the model was trained using a dataset of compounds with proven antibacterial properties. This ensured that key features of antibiotics were incorporated into its decision-making process. However, this approach also limited the model’s ability to predict novel antibacterial compounds, particularly those with structures or mechanisms distinct from those in the training set. To enhance the model’s performance, additional data should be incorporated into the retraining process. This could include an up-to-date set of antibacterial compounds from the continuously updated database, for example, Pubchem [24], ChEMBL [43], AntibioticDB [44]. Although the model achieved high predictive performance across antibiotic classes, some classes were represented by only a small number of compounds in the dataset (S1 Fig). Therefore, the performance estimates for these classes should be interpreted with caution, as their structural diversity may not be adequately represented. Future studies should incorporate additional compounds from underrepresented classes to improve model robustness and generalizability. However, even after dataset expansion, some degree of imbalance may persist. Therefore, strategies tailored for imbalanced data—such as data-level resampling methods (e.g., oversampling of minority classes) and the use of class-weighted learning algorithms—will be implemented during model retraining. These approaches are expected to enhance class-specific predictive performance and improve overall model generalizability.

## Conclusion

In our study, a model for predicting the antibacterial properties of compounds was developed. Data on compounds with and without antibacterial properties were downloaded from the PubChem database and manually curated. Chemometric descriptors of both classes were generated with the RDKit package, and then used to train and test the algorithm. After fine-tuning and feature selection, the predictive performance was high, with accuracy, precision, sensitivity, and AUC-ROC exceeding 0.9. The model also showed good accuracy toward almost all classes of antibiotics. The fully trained model is freely available in a public database and should be beneficial for researchers interested in antibiotic discovery. The model is available at: https://antibacterial-predictor-model-ocogzqyibervrqb7trvfev.streamlit.app/.

## Supporting information

S1 Fig Performance of the fully developed model across nine classes of antibiotics. The numbers next to each bar in the histogram indicate the model’s accuracy for the corresponding antibiotic class. The numbers in parentheses represent the number of antibiotics used to calculate the accuracy for that class.
(TIFF)

S1 Table Raw data for machine learning algorithm development. (XLSX)
