# Supplementary material for: Machine learning-based quantitative structure–activity relationship model for antibiotic prediction and discovery
Source: PLOS Digit Health. 2026 Aug 13;5(8):e0001593. doi: 10.1371/journal.pdig.0001593 (PMC13472409; doi:10.1371/journal.pdig.0001593)
Supplement: Supplementary file 1 [file pdig.0001593.s004.docx]

**Response to reviewers**

Firstly, we would like to thank the editor and all reviewers for the valuable suggestion which will improve the quality of our manuscript. According to your suggestion, we tried to respond and correct as much as we could. Please find them below.

**Reviewer #1**

Comment 1: The article states that hyperparameter tuning did not produce any improvement in DT model performance (accuracy, precision, and sensitivity all decreased slightly by 0.01)

Response: We thank the reviewer for the insightful comment. We performed hyperparameter tuning using K-fold cross-validation; however, a slight decrease in model performance (approximately 0.01) was observed. Cases in which hyperparameter tuning does not improve model performance—and may even reduce it—have been reported previously. For example, Probst et al. (2019) conducted a large-scale benchmarking study to assess the tunability of common machine learning algorithms and demonstrated that tuning does not consistently lead to performance improvements. Similarly, Mantovani et al. (2024) investigated the effects of hyperparameter tuning in two widely used decision tree induction algorithms and found that tuning improved the performance of the C4.5 model in only about one-third of the evaluated cases. Therefore, the unimproved performance observed after tuning in our study is not unexpected and is consistent with observations reported in earlier studies.

References

- Mantovani RG, Horváth T, Rossi ALD, Vanschoren J, de Carvalho ACPLF. Better trees: An empirical study on hyperparameter tuning of classification decision tree induction algorithms. *Data Min Knowl Discov.* 2024;38:1364-1416. doi:10.1007/s10618-024-01002-5
- Probst P, Boulesteix AL, Bischl B. Tunability: Importance of hyperparameters of machine learning algorithms. *J Mach Learn Res*. 2019;20(53):1-32.

Comment 2: It is poorly documented why specific parameters were chosen to tune (e.g., $Log_{2}=0.5$ and 0.75, which seems to be a typo for a feature fraction).

Response: The hyperparameters used for tuning were selected based on the recommended settings of the default decision tree model. We also apologize for the typo error in the original manuscript and have corrected it in the revised version (Line 190-191).

Comment 3: While the feature selection is a positive direction towards interpretability and efficiency, the results reporting is somewhat disconnected. The change in the feature importance ranking when selected could be discussed more integrally with the chemical relevance in the main Results section rather than waiting until the Feature Analysis and Discussion section.

Response: We agree that placing the feature analysis immediately after the feature selection step improves the logical flow and strengthens the connection between the findings. Accordingly, we have removed the section header and relocated the feature analysis text within the Results section (Lines 292–293).

After careful consideration, we chose not to expand the discussion on chemical relevance within the Results section, as doing so could distract readers from the main methodological and predictive insights presented. We believe that the current structure allows readers to more clearly follow the rationale and outcomes of our analysis, while leaving detailed chemical interpretation to the Discussion section.

Comment 4: The low accuracy for the sulfonamide class is rightly discussed as a limitation, possibly due to low representation (0.49%). This necessitates a more detailed discussion or a clear mention about how future work will address this data imbalance.

Response: We have added suggestions for future work to address data imbalance in certain drug classes, as suggested by the reviewer (Line 457-461).

Comment 5: State which two algorithms performed similarly in the abstract (DT and SVM) 16. The current text is "DT and SVM algorithms showed comparable performance" which is okay, but a minor change for best clarity is suggested.

Response: The Abstract has been revised as suggested by the reviewer (Line 35-37).

Comment 6: In the Feature Analysis section, include a concise table or bulleted list that clearly separates the top 20 descriptors by their descriptor type (T, C, E, P, MD) and then immediately follow it with a more integrated discussion of their chemical pertinence. The current approach of scattering the explanation across makes it harder to read.

Response: Thank you for your consideration on this matter. We have reallocated the feature analysis section as described in our response to Comment 3, with the aim of improving clarity and reader understanding. We chose not to add an additional table or bullet list, as this information would be redundant with Table 3. After careful evaluation, we believe that the revised manuscript is logically structured and clearly presented.

Comment 7: Current coverage of the machine learning algorithms in the application area of drug discovery, QSAR, is good. It could be better if it cites a broader perspective of how ML and IoT intersect because this QSAR study includes a digital health approach and computer-based methods (the likes of a smart system).

Response: We agree that the intersection of machine learning and IoT for developing smart systems in downstream digital health applications is both beneficial and of interest. However, the primary scope of this study is the development of a computational tool to support upstream drug discovery processes. Including IoT-related aspects in the current manuscript may divert reader focus and introduce potential confusion regarding the intended application and position of the proposed method within the overall discovery pipeline. Therefore, we have decided not to include IoT-related discussions in this manuscript.

Comment 8: It is suggested to establish the connection of machine learning with this research's digital/intelligent systems context stronger in the Introduction or Discussion section. A good position would be following the sentence of machine learning algorithms in QSAR. The suggested paper from the provided below is appropriate to elicit the overall background of ML in intelligent systems: Reference to Add: Systematic Analysis based on Conflux of Machine Learning and Internet of Things using Bibliometric analysis. The suggested reference to refer is: "Systematic analysis based on Conflux of machine learning and internet of things using bibliometric analysis".

Response: We have reviewed the suggested publication and found that it outlines several interesting aspects of IoT applications across various fields, including healthcare. However, the publication does not address drug discovery or computational drug screening, which are the primary focus of our manuscript. As such, we were unable to identify an appropriate context in which to cite this work. Nevertheless, we will consider this reference in future studies where its scope is more directly relevant.

**Reviewer #2**

Comment 1: The novelty is limited as the general antibacterial QSAR classifiers have been already reported.

Response: While several previous studies have employed pipelines similar to ours, as summarized in Table 4, this work offers clear advantages that address practical needs in early-stage antibacterial discovery. In particular, our model focuses on general bacterial activity rather than species-specific targets, making it well suited for primary screening of antibacterial compounds. We also adopted explainable machine learning algorithms to improve the transparency and interpretability of the prediction process, which helps build confidence in the model outputs. In addition, we made the trained model publicly available to encourage broader use and reuse. Overall, we believe this work provides a practical and useful resource for the research community and can meaningfully support antibacterial drug discovery efforts.

Comment 2: The authors should more clearly highlight the unique contributions of this work, such as dataset scale, interpretability, and model deployment.

Response: We agreed that contributions of this work should be acknowledged in the manuscript, hence, we added suggested strength of this work into the discussion part (Line 431-439).

Comment 3: The positive dataset consist of known antibiotic compounds, while the negative dataset contains different drug classes.

Response: We intentionally included multiple classes of compounds in the negative dataset to increase molecular diversity and support more effective model learning. If the negative dataset were limited to a single compound class, such as nonsteroidal anti-inflammatory drugs (NSAIDs), the model would mainly learn to distinguish antibiotics from that specific class rather than capturing general features related to antibacterial activity. As a result, its performance could be compromised when applied to compounds from other chemical classes.

Because compounds without antibacterial activity greatly outnumber active compounds in practice, we therefore selected a representative subset of negative compounds and balanced their number with the positive dataset to avoid class imbalance during training. We expected that this balanced and chemically diverse design would help the model generalize better to a wide range of compound structures likely to be encountered by users in future applications, while also reducing technical bias associated with imbalanced data.

Comment 4: This design might help to recognise the “antibiotic-like”, rather than true antibacterial activity.

Response: This is an excellent point and well worth noting. We have added this aspect to the limitations of the study and emphasized the importance of biological validation of the model predictions (Line 441-446).

Comment 5: The parameters used for defining anti-bacterial activity are not explained in detail. (e.g. assay selection, MIC)

Response: We apologize for the confusion regarding the definition of antibacterial activity in the original manuscript. We have clarified this point in the revised Methods section by explaining the criteria used to define the positive dataset, including the use of MIC (Lines 153–156).

Comment 6: Dataset construction for predicting structurally novel antibacterial compounds should be discussed in detail.

Response: Information on continuously updated chemical compound databases, including those containing antibacterial activity data, has been added to the Discussion section as suggested, to support dataset construction for training models aimed at predicting novel antibacterial compounds (Line 452-453).

Comment 7: The author should give limitation and future directions

Response: The limitations and future directions were added to the revised manuscript (Line 439-461).

Comment 8: The rationale for algorithm selection has to be clarified.

Response: We chose DT, SVM, and NB because they were commonly used in QSAR modeling, as reported in Table 4 and from the review of Keyvanpour and Shirzad (2021). From all of the models, DT was chosen for its interpretability, SVM for its effectiveness in high-dimensional feature spaces, and NB as a simple probabilistic baseline, enabling a balanced and systematic comparison of different modeling approaches. This information was added to the method section in the revised version of the manuscript (Line 178-181).

Reference

- Keyvanpour MR, Shirzad MB. An Analysis of QSAR Research Based on Machine Learning Concepts. *Curr Drug Discov Technol*. 2021;18(1):17-30. doi:10.2174/1570163817666200316104404

Comment 9: The model shows reduced predictive performance for sulphonamide antibiotics.
While this makes class imbalance, the issue highlights a limitation of the dataset.

Response: The underrepresentation of sulfonamide drugs was listed as a limitation of the study, and potential future directions to address this issue were suggested (Line 457-461).

Comment 10: The number of drug per antibiotic class should be reported.

Response: We reported the number of drugs in each class in the test set, as shown in S1 Fig, to illustrate model performance across different classes of known antibiotics. We did not perform the same analysis for the training set, as this would be labor-intensive and would not provide additional insight into model performance. Because the dataset was randomly divided into training and test sets, the distribution of antibiotic classes in the training set is expected to be similar to that observed in the test set.

Comment 11: Figure 5 can be more clear

Response: Due to the complexity and level of detail involved in the model decision process, this figure may be difficult to interpret. We have therefore rearranged the figure to improve clarity and enhance reader understanding.

Comment 12: Some references appear unnecessary and could be consolidated

Response: We have reviewed the references and removed those that were less essential to the manuscript, consolidating the remaining citations accordingly to improve clarity (Lines 119–121).
