# Supplementary material for: Machine learning-based quantitative structure–activity relationship model for antibiotic prediction and discovery
Source: PLOS Digit Health. 2026 Aug 13;5(8):e0001593. doi: 10.1371/journal.pdig.0001593 (PMC13472409; doi:10.1371/journal.pdig.0001593)
Supplement: Supplementary file 2 [file pdig.0001593.s005.docx]

**Response to reviewer**

We would like to sincerely thank the reviewer for the careful re-evaluation of our revised manuscript and for the constructive feedback. We have carefully addressed the remaining comments and made additional revisions accordingly. Our detailed responses are provided below.

**Reviewer #1**

Comment 1: The algorithms used, such as Decision Trees, Support Vector Machines, and Naive Bayes, are common in this field. While they work well, the manuscript does not compare these methods to newer deep learning models like Graph Convolutional Networks, which are often used for molecular data.

Response: We thank the Reviewer for this valuable comment. We agree that deep learning approaches, including Graph Convolutional Networks (GCNs), have shown strong performance in molecular property prediction and antibacterial discovery. However, the classical machine-learning models such as Decision Trees, Support Vector Machines, and Naïve Bayes offer advantages in terms of interpretability, lower computational requirements, and ease of deployment as a user-friendly web application. Therefore, we included the 3 models in our present study. The rationale for model selection was stated in material and method section (Line 172-175).

Comment 2: The model does not perform well on sulfonamides, achieving an accuracy of less than 80%. This is likely due to their low representation in the dataset, making up only 0.49% of the data.

Response: We agree that the reduced predictive performance for sulfonamides was likely attributable to their low representation in the dataset, which limited the model's ability to learn class-specific structural patterns. Upon retraining the model, the predictive accuracy for the sulfonamide class improved to 100%. However, we recognize that this apparent improvement may also be influenced by the limited number of sulfonamide compounds available for evaluation. Therefore, we continue to acknowledge the low representation of certain antibiotic classes as a limitation of the study, and corresponding future directions to address this issue have been proposed in the revised manuscript. (Line 459-469).

Comment 3: The model mostly identifies molecules that resemble known antibiotics rather than discovering truly new biological mechanisms.

Response: We appreciate this insightful comment. We agree that supervised learning models trained on known antibiotics may preferentially identify compounds with structural similarity to existing antibacterial scaffolds, rather than uncover entirely new biological mechanisms. This limitation and its future direction were stated in the manuscript (Line 441-447).

Comment 4: There were technical typos in the original hyperparameter settings, but the authors say they have fixed these in the revised version.

Response: The technical typos in the original submission have been corrected in the revised manuscript (Line 184-185). We thank the Reviewer for bringing these issues to our attention.

Comment 5: The authors need to clearly outline how they will retrain the model. This should include strategies like oversampling or gathering more data for drug classes like sulfonamides, which are underrepresented.

Response: We thank the Reviewer for highlighting this important point. We have revised the manuscript to include a more detailed description of the model retraining process in the limitation of the study, with particular attention to the underrepresentation of the sulfonamide class (Line 465-469).

Comment 6: To move beyond just recognizing "antibiotic-like" structures, the authors should suggest a plan for testing their model's predictions in laboratory experiments.

Response: The plan for experimental validation of the predicted antibacterial compounds has been added to the revised version of the manuscript (Line 444-447).

Comment 7: Although the feature analysis has been moved to the Results section, it’s important to ensure that the transition between method insights and chemical interpretations is smooth to avoid confusing the reader.

Response: The bridging sentence was added to ensure smooth transition between paragraph presenting chemical information and the antibiotic class performances in the revised manuscript (Line 306-307).

Comment 8: Ensure the link to the Python package works and that the documentation is easy to understand for non-data scientists. Keep the detailed discussion on the importance of β-lactams, as it provides important context for the model’s success.

Response: Thank you for this important point. Upon re-evaluating the Python package, we found that the original implementation contained issues that could not be reliably resolved through minor corrections. Therefore, we decided to rebuild the tool and retrain the model from the beginning. Reassuringly, the newly trained model achieved performance comparable to that reported in the original manuscript, demonstrating the reproducibility and robustness of our workflow. Although there were minor changes in the ranking of feature importance, the key descriptors remained largely consistent and continued to support the biological and chemical rationale underlying the model predictions.

In addition, the retrained model showed improved predictive performance across all antibiotic classes. During this process, we also reconsidered the deployment strategy of the tool. Rather than distributing it as a Python package, which requires users to install dependencies and have basic programming experience, we developed a web application that can be accessed directly through a web browser. This approach improves accessibility and usability, particularly for researchers without a data science or programming background. The web application has been tested and is functioning properly, and the user documentation has been revised to further improve clarity and ease of use.

We have also kept the discussion on the importance of β-lactams, as it provides important context for interpreting the model’s performance. Despite the improved results, we continue to acknowledge the limited representation of some antibiotic classes in the training dataset as a study limitation and have retained the corresponding discussion in the revised manuscript.
