# Supplementary material for: Machine learning-based quantitative structure–activity relationship model for antibiotic prediction and discovery
Source: PLOS Digit Health. 2026 Aug 13;5(8):e0001593. doi: 10.1371/journal.pdig.0001593 (PMC13472409; doi:10.1371/journal.pdig.0001593)
Supplement: Peer review history

# Peer review: Decision Letter 0

**DOI:** 10.1371/journal.pdig.0001593.r001

**Authors:** Dukyong Yoon

6 Jan 2026

PDIG-D-25-00474

Machine learning-based quantitative structure–activity relationship model for antibiotic prediction and discovery

PLOS Digital Health

Dear Dr. Chienwichai,

Thank you for submitting your manuscript to PLOS Digital Health. After careful consideration, we feel that it has merit but does not fully meet PLOS Digital Health's publication criteria as it currently stands. Therefore, we invite you to submit a revised version of the manuscript that addresses the points raised during the review process.

Please submit your revised manuscript by Feb 05 2026 11:59PM. If you will need more time than this to complete your revisions, please reply to this message or contact the journal office at digitalhealth@plos.org. When you're ready to submit your revision, log on to https://www.editorialmanager.com/pdig/ and select the 'Submissions Needing Revision' folder to locate your manuscript file.

Please include the following items when submitting your revised manuscript:

* A letter that responds to each point raised by the editor and reviewer(s). You should upload this letter as a separate file labeled 'Response to Reviewers'. This file does not need to include responses to any formatting updates and technical items listed in the 'Journal Requirements' section below.

* A marked-up copy of your manuscript that highlights changes made to the original version. You should upload this as a separate file labeled 'Revised Manuscript with Track Changes'.

* An unmarked version of your revised paper without tracked changes. You should upload this as a separate file labeled 'Manuscript'.

If you would like to make changes to your financial disclosure, competing interests statement, or data availability statement, please make these updates within the submission form at the time of resubmission. Guidelines for resubmitting your figure files are available below the reviewer comments at the end of this letter.

We look forward to receiving your revised manuscript.

Kind regards,

Dukyong Yoon

Section Editor

PLOS Digital Health

Gilles Guillot

Academic Editor

PLOS Digital Health

Leo Anthony Celi

Editor-in-Chief

PLOS Digital Health

orcid.org/0000-0001-6712-6626

**Journal Requirements:**

1. We have amended your Competing Interest statement to comply with journal style. We kindly ask that you double check the statement and let us know if anything is incorrect.

2. If the reviewer comments include a recommendation to cite specific previously published works, please review and evaluate these publications to determine whether they are relevant and should be cited. There is no requirement to cite these works unless the editor has indicated otherwise.

[Note: HTML markup is below. Please do not edit.]

**Reviewers' Comments:**

Reviewer's Responses to Questions

**Comments to the Author**

1. Does this manuscript meet PLOS Digital Health’s publication criteria? Is the manuscript technically sound, and do the data support the conclusions? The manuscript must describe methodologically and ethically rigorous research with conclusions that are appropriately drawn based on the data presented.

Reviewer #1: Yes

Reviewer #2: Yes

**********

2. Has the statistical analysis been performed appropriately and rigorously?

Reviewer #1: Yes

Reviewer #2: Yes

**********

3. Have the authors made all data underlying the findings in their manuscript fully available (please refer to the Data Availability Statement at the start of the manuscript PDF file)?

The PLOS Data policy requires authors to make all data underlying the findings described in their manuscript fully available without restriction, with rare exception. The data should be provided as part of the manuscript or its supporting information, or deposited to a public repository. For example, in addition to summary statistics, the data points behind means, medians and variance measures should be available. If there are restrictions on publicly sharing data—e.g. participant privacy or use of data from a third party—those must be specified.

Reviewer #1: Yes

Reviewer #2: Yes

**********

4. Is the manuscript presented in an intelligible fashion and written in standard English?

PLOS Digital Health does not copyedit accepted manuscripts, so the language in submitted articles must be clear, correct, and unambiguous. Any typographical or grammatical errors should be corrected at revision, so please note any specific errors here.

Reviewer #1: Yes

Reviewer #2: Yes

**********

5. Review Comments to the Author

Please use the space provided to explain your answers to the questions above. You may also include additional comments for the author, including concerns about dual publication, research ethics, or publication ethics. (Please upload your review as an attachment if it exceeds 20,000 characters)

Reviewer #1: 1. The article states that hyperparameter tuning did not produce any improvement in DT model performance (accuracy, precision, and sensitivity all decreased slightly by 0.01)10. It is poorly documented why specific parameters were chosen to tune (e.g., $Log_{2}=0.5$ and 0.75, which seems to be a typo for a feature fraction)11.

2. While the feature selection is a positive direction towards interpretability and efficiency, the results reporting is somewhat disconnected. The change in the feature importance ranking when selected could be discussed more integrally with the chemical relevance in the main Results section rather than waiting until the Feature Analysis and Discussion section.

3. The low accuracy for the sulfonamide class is rightly discussed as a limitation, possibly due to low representation (0.49%). This necessitates a more detailed discussion or a clear mention about how future work will address this data imbalance14.

4. State which two algorithms performed similarly in the abstract (DT and SVM)16. The current text is "DT and SVM algorithms showed comparable performance" which is okay, but a minor change for best clarity is suggested.

5. In the Feature Analysis section, include a concise table or bulleted list that clearly separates the top 20 descriptors by their descriptor type (T, C, E, P, MD) and then immediately follow it with a more integrated discussion of their chemical pertinence. The current approach of scattering the explanation across makes it harder to read.

6. Current coverage of the machine learning algorithms in the application area of drug discovery, QSAR, is good. It could be better if it cites a broader perspective of how ML and IoT intersect because this QSAR study includes a digital health approach and computer-based methods (the likes of a smart system).

7. It is suggested to establish the connection of machine learning with this research's digital/intelligent systems context stronger in the Introduction or Discussion section. A good position would be following the sentence of machine learning algorithms in QSAR. The suggested paper from the provided below is appropriate to elicit the overall background of ML in intelligent systems: Reference to Add: Systematic Analysis based on Conflux of Machine Learning and Internet of Things using Bibliometric analysis. The suggested reference to refer is: "Systematic analysis based on Conflux of machine learning and internet of things using bibliometric analysis".

Reviewer #2: The manuscript addresses the important problem in AMR using machine learning-based QSAR modelling. The study is technically sound and clearly written, also providing model with available python package.

1.The novelty is limited as the general antibacterial QSAR classifiers have been already reported.

2. The authors should more clearly highlight the unique contributions of this work, such as dataset scale, interpretability, and model deployment.

3. The positive dataset consist of known antibiotic compounds, while the negative dataset contains different drug classes.

4.This design might help to recognise the “antibiotic-like”, rather than true antibacterial activity.

5.The parameters used for defining anti-bacterial activity are not explained in detail. (e.g. assay selection, MIC)

6.Dataset construction for predicting structurally novel antibacterial compounds should be discussed in detail

7.The author should give limitation and future directions

8.The rationale for algorithm selection has to be clarified.

9. The model shows reduced predictive performance for sulphonamide antibiotics.

While this makes class imbalance, the issue highlights a limitation of the dataset.

10.The number of drug per antibiotic class should be reported.

11.Figure 5 can be more clear

12.Some references appear unnecessary and could be consolidated

**********

6. PLOS authors have the option to publish the peer review history of their article (what does this mean?). If published, this will include your full peer review and any attached files.

**Do you want your identity to be public for this peer review?**  If you choose “no”, your identity will remain anonymous but your review may still be made public.

For information about this choice, including consent withdrawal, please see our Privacy Policy.

Reviewer #1: No

Reviewer #2: No

**********

[NOTE: If reviewer comments were submitted as an attachment file, they will be attached to this email and accessible via the submission site. Please log into your account, locate the manuscript record, and check for the action link "View Attachments". If this link does not appear, there are no attachment files.]

**Figure resubmission:**

While revising your submission, we strongly recommend that you use PLOS’s NAAS tool (https://ngplosjournals.pagemajik.ai/artanalysis) to test your figure files. NAAS can convert your figure files to the TIFF file type and meet basic requirements (such as print size, resolution), or provide you with a report on issues that do not meet our requirements and that NAAS cannot fix.

After uploading your figures to PLOS’s NAAS tool - https://ngplosjournals.pagemajik.ai/artanalysis, NAAS will process the files provided and display the results in the "Uploaded Files" section of the page as the processing is complete. If the uploaded figures meet our requirements (or NAAS is able to fix the files to meet our requirements), the figure will be marked as "fixed" above. If NAAS is unable to fix the files, a red "failed" label will appear above. When NAAS has confirmed that the figure files meet our requirements, please download the file via the download option, and include these NAAS processed figure files when submitting your revised manuscript.

**Reproducibility:**

To enhance the reproducibility of your results, we recommend that authors of applicable studies deposit laboratory protocols in protocols.io, where a protocol can be assigned its own identifier (DOI) such that it can be cited independently in the future. Additionally, PLOS ONE offers an option to publish peer-reviewed clinical study protocols. Read more information on sharing protocols at https://plos.org/protocols?utm_medium=editorial-email&utm_source=authorletters&utm_campaign=protocols

# Author comment: Author response to Decision Letter 1

**DOI:** 10.1371/journal.pdig.0001593.r002

2 Feb 2026

Attachment Submitted filename: Responses to reviewers.docx

# Peer review: Decision Letter 1

**DOI:** 10.1371/journal.pdig.0001593.r003

**Authors:** Dukyong Yoon

26 Feb 2026

PDIG-D-25-00474R1

Machine learning-based quantitative structure–activity relationship model for antibiotic prediction and discovery

PLOS Digital Health

Dear Dr. Chienwichai,

Thank you for submitting your manuscript to PLOS Digital Health. After careful consideration, we feel that it has merit but does not fully meet PLOS Digital Health's publication criteria as it currently stands. Therefore, we invite you to submit a revised version of the manuscript that addresses the points raised during the review process.

Please submit your revised manuscript by Apr 27 2026 11:59PM. If you will need more time than this to complete your revisions, please reply to this message or contact the journal office at digitalhealth@plos.org. When you're ready to submit your revision, log on to https://www.editorialmanager.com/pdig/ and select the 'Submissions Needing Revision' folder to locate your manuscript file.

Please include the following items when submitting your revised manuscript:

* A letter that responds to each point raised by the editor and reviewer(s). You should upload this letter as a separate file labeled 'Response to Reviewers'. This file does not need to include responses to any formatting updates and technical items listed in the 'Journal Requirements' section below.

* A marked-up copy of your manuscript that highlights changes made to the original version. You should upload this as a separate file labeled 'Revised Manuscript with Track Changes'.

* An unmarked version of your revised paper without tracked changes. You should upload this as a separate file labeled 'Manuscript'.

If you would like to make changes to your financial disclosure, competing interests statement, or data availability statement, please make these updates within the submission form at the time of resubmission. Guidelines for resubmitting your figure files are available below the reviewer comments at the end of this letter.

We look forward to receiving your revised manuscript.

Kind regards,

Dukyong Yoon

Section Editor

PLOS Digital Health

Dukyong Yoon

Section Editor

PLOS Digital Health

Leo Anthony Celi

Editor-in-Chief

PLOS Digital Health

orcid.org/0000-0001-6712-6626

**Journal Requirements:**

If the reviewer comments include a recommendation to cite specific previously published works, please review and evaluate these publications to determine whether they are relevant and should be cited. There is no requirement to cite these works unless the editor has indicated otherwise.

**Additional Editor Comments (if provided):**

[Note: HTML markup is below. Please do not edit.]

**Reviewers' Comments:**

Reviewer's Responses to Questions

**Comments to the Author**

1. If the authors have adequately addressed your comments raised in a previous round of review and you feel that this manuscript is now acceptable for publication, you may indicate that here to bypass the “Comments to the Author” section, enter your conflict of interest statement in the “Confidential to Editor” section, and submit your "Accept" recommendation.

Reviewer #1: (No Response)

**********

2. Does this manuscript meet PLOS Digital Health’s publication criteria? Is the manuscript technically sound, and do the data support the conclusions? The manuscript must describe methodologically and ethically rigorous research with conclusions that are appropriately drawn based on the data presented.

Reviewer #1: Yes

**********

3. Has the statistical analysis been performed appropriately and rigorously?

Reviewer #1: Yes

**********

4. Have the authors made all data underlying the findings in their manuscript fully available (please refer to the Data Availability Statement at the start of the manuscript PDF file)?

The PLOS Data policy requires authors to make all data underlying the findings described in their manuscript fully available without restriction, with rare exception. The data should be provided as part of the manuscript or its supporting information, or deposited to a public repository. For example, in addition to summary statistics, the data points behind means, medians and variance measures should be available. If there are restrictions on publicly sharing data—e.g. participant privacy or use of data from a third party—those must be specified.

Reviewer #1: Yes

**********

5. Is the manuscript presented in an intelligible fashion and written in standard English?

PLOS Digital Health does not copyedit accepted manuscripts, so the language in submitted articles must be clear, correct, and unambiguous. Any typographical or grammatical errors should be corrected at revision, so please note any specific errors here.

Reviewer #1: Yes

**********

6. Review Comments to the Author

Please use the space provided to explain your answers to the questions above. You may also include additional comments for the author, including concerns about dual publication, research ethics, or publication ethics. (Please upload your review as an attachment if it exceeds 20,000 characters)

Reviewer #1: 1. The algorithms used, such as Decision Trees, Support Vector Machines, and Naive Bayes, are common in this field. While they work well, the manuscript does not compare these methods to newer deep learning models like Graph Convolutional Networks, which are often used for molecular data.

2. The model does not perform well on sulfonamides, achieving an accuracy of less than 80%. This is likely due to their low representation in the dataset, making up only 0.49% of the data.

3. The model mostly identifies molecules that resemble known antibiotics rather than discovering truly new biological mechanisms.

4. There were technical typos in the original hyperparameter settings, but the authors say they have fixed these in the revised version.

5. The authors need to clearly outline how they will retrain the model. This should include strategies like oversampling or gathering more data for drug classes like sulfonamides, which are underrepresented.

6. To move beyond just recognizing "antibiotic-like" structures, the authors should suggest a plan for testing their model's predictions in laboratory experiments.

7. Although the feature analysis has been moved to the Results section, it’s important to ensure that the transition between method insights and chemical interpretations is smooth to avoid confusing the reader.

8. Ensure the link to the Python package works and that the documentation is easy to understand for non-data scientists. Keep the detailed discussion on the importance of β-lactams, as it provides important context for the model’s success. Once revised, this manuscript has great potential to be a valuable resource for researchers looking for fast, clear screening methods to combat antimicrobial resistance.

**********

7. PLOS authors have the option to publish the peer review history of their article (what does this mean?). If published, this will include your full peer review and any attached files.

**Do you want your identity to be public for this peer review?**  If you choose “no”, your identity will remain anonymous but your review may still be made public.

For information about this choice, including consent withdrawal, please see our Privacy Policy.

Reviewer #1: No

**********

[NOTE: If reviewer comments were submitted as an attachment file, they will be attached to this email and accessible via the submission site. Please log into your account, locate the manuscript record, and check for the action link "View Attachments". If this link does not appear, there are no attachment files.]

**Figure resubmission:**

While revising your submission, we strongly recommend that you use PLOS’s NAAS tool (https://ngplosjournals.pagemajik.ai/artanalysis) to test your figure files. NAAS can convert your figure files to the TIFF file type and meet basic requirements (such as print size, resolution), or provide you with a report on issues that do not meet our requirements and that NAAS cannot fix.

After uploading your figures to PLOS’s NAAS tool - https://ngplosjournals.pagemajik.ai/artanalysis, NAAS will process the files provided and display the results in the "Uploaded Files" section of the page as the processing is complete. If the uploaded figures meet our requirements (or NAAS is able to fix the files to meet our requirements), the figure will be marked as "fixed" above. If NAAS is unable to fix the files, a red "failed" label will appear above. When NAAS has confirmed that the figure files meet our requirements, please download the file via the download option, and include these NAAS processed figure files when submitting your revised manuscript.

**Reproducibility:**

To enhance the reproducibility of your results, we recommend that authors of applicable studies deposit laboratory protocols in protocols.io, where a protocol can be assigned its own identifier (DOI) such that it can be cited independently in the future. Additionally, PLOS ONE offers an option to publish peer-reviewed clinical study protocols. Read more information on sharing protocols at https://plos.org/protocols?utm_medium=editorial-email&utm_source=authorletters&utm_campaign=protocols

# Author comment: Author response to Decision Letter 2

**DOI:** 10.1371/journal.pdig.0001593.r004

2 Jun 2026

Attachment Submitted filename: Responses to reviewers - R1.docx

# Editor report: Decision Letter 2

**DOI:** 10.1371/journal.pdig.0001593.r005

**Authors:** Dukyong Yoon

5 Jul 2026

Machine learning-based quantitative structure–activity relationship model for antibiotic prediction and discovery

PDIG-D-25-00474R2

Dear Associate professor Chienwichai,

We are pleased to inform you that your manuscript 'Machine learning-based quantitative structure–activity relationship model for antibiotic prediction and discovery' has been provisionally accepted for publication in PLOS Digital Health.

Before your manuscript can be formally accepted you will need to complete some formatting changes, which you will receive in a follow-up email from a member of our team.

Please note that your manuscript will not be scheduled for publication until you have made the required changes, so a swift response is appreciated.

IMPORTANT: The editorial review process is now complete. PLOS will only permit corrections to spelling, formatting or significant scientific errors from this point onwards. Requests for major changes, or any which affect the scientific understanding of your work, will cause delays to the publication date of your manuscript.

If your institution or institutions have a press office, please notify them about your upcoming paper to help maximize its impact. If they'll be preparing press materials, please inform our press team as soon as possible -- no later than 48 hours after receiving the formal acceptance. Your manuscript will remain under strict press embargo until 2 pm Eastern Time on the date of publication. For more information, please contact digitalhealth@plos.org.

Thank you again for supporting Open Access publishing; we are looking forward to publishing your work in PLOS Digital Health.

Best regards,

Dukyong Yoon

Section Editor

PLOS Digital Health

***********************************************************

**Additional Editor Comments (if provided):**

**Reviewer Comments (if any, and for reference):**
